# Amelogenesis imperfecta with Class III malocclusion, reduced crown size and decreased OVD: A multi‐disciplinary management and a 5‐year follow‐up

**DOI:** 10.1002/ccr3.2874

**Published:** 2020-05-07

**Authors:** Amel Labidi, Sana Bekri, Yosra Mabrouk, Jouda Ben Mustapha, Monia Omezzine, Sonia Ghoul‐Mazgar, Lamia Mansour

**Affiliations:** ^1^ Department of Removable Prosthodontics Faculty of Dental Medicine University of Monastir Monastir Tunisia; ^2^ ABCDF Laboratory for Biological Clinical and Dento‐Facial Approach University of Monastir Monastir Tunisia; ^3^ Private Practice Tunis Tunisia; ^4^ Department of Maxillofacial, Plastic and Aesthetic Surgery Sahloul University Hospital Sousse Tunisia; ^5^ Laboratory of Dento-Facial, Clinical and Biological Approach (ABCDF) Faculty of Dental Medicine University of Monastir Monastir Tunisia

**Keywords:** amelogenesis Imperfecta, crown lengthening, Lefort I osteotomy, metal‐ceramic restorations, vertical dimension

## Abstract

This clinical report describes the oral rehabilitation of a 22‐year‐old‐man diagnosed with a variant of hypoplastic amelogenesis imperfecta. The treatment approach was multi‐disciplinary, and it included the surgical procedure of Lefort I osteotomy, surgical crown lengthening, and metal‐ceramic‐fixed dental prostheses. The patient was satisfied with the esthetic and functional outcome.

## Graphical abstract

Amelogenesis imperfecta cases are often complex regarding the altered dental structure and the oro‐facial anomalies. Their treatment requires a reorganized approach through an interdisciplinary collaboration to assess optimal esthetics and function.

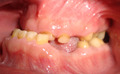

## 1 INTRODUCTION

Amelogenesis imperfecta (AI) is a dental anomaly including a group of hereditary disorders that affect the quality and/or the quantity of enamel in both primary and permanent dentitions. Both forms of AI, isolated and syndromic, are described in the literature.^1^ The inheritance pattern of AI may be autosomal dominant, autosomal recessive, or X‐linked.^2^ Its prevalence varies between 1/700^3^ and 1/14000.^4^ According to Witkop classification, revised by Nusier et al, there are four main forms of AI: hypoplastic, hypocalcified, hypomatured, and AI with taurodontism.^5^ Concerning the cranio‐facial features, AI is known not only for the structure anomalies of the enamel but also for its frequent association with a reduction of the crown size, agenesis, and extensive loss of tooth tissues, leading to the loss of occlusal vertical dimension (OVD).^6^ Besides, AI has been shown to be associated with skeletal anomalies such as open bite and skeletal Class II or Class III malocclusions.^1^

The oro‐facial and dental treatment planning depends on the severity of A.I cases. While some patients may be treated using conservative methods, it is a challenge for clinicians to achieve an esthetic and functional restoration together with stable occlusion in most severe cases. ^1^ A multi‐disciplinary approach involving surgery, orthodontics, periodontology, and prosthodontics is required for such cases.^7^

The aim of this paper was to present a multi‐disciplinary approach to a patient diagnosed with hypoplastic AI associated with Class III skeletal malocclusion, reduced crown size and decreased OVD.

## 2 CASE REPORT

**Figure 1:**
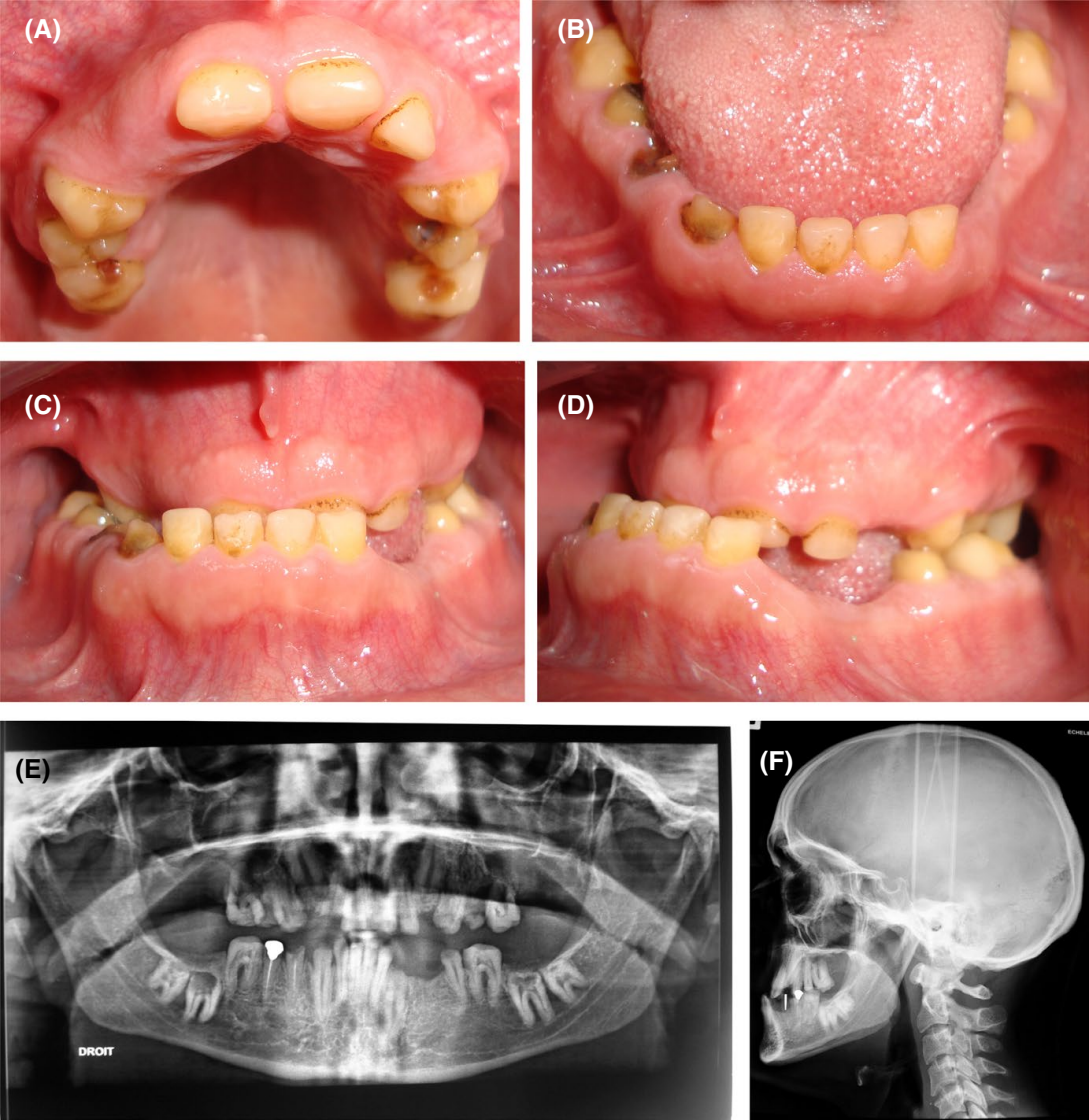
Initial clinical data. A, Occlusal view of the maxillary arch. B, Pretreatment occlusal view of the mandibular arch. C, Intraoral front view showing a reverse articulation in the bilateral posterior regions and a decreased vertical dimension. D, Intraoral right lateral view underlying reverse articulation in anterior region and the short height of teeth. E, orhopantomographic radiograph. F, Lateral cephalogram radiograph

A 22‐year‐old man was referred to the Prosthodontics Department to improve his poor smile esthetics as well as his chewing ability. No remarkable findings were identified in his medical record. The extraoral examination revealed facial asymmetry on the patient's front view. However, from a side view, a lower lip and chin protrusion were noticed. Intraoral examination (Figure 1A, B) showed permanent teeth with yellow discoloration and enamel smooth surfaces, diastemas between the teeth, and short as well as deformed clinical crowns. In occlusion, anterior and posterior cross bites and a decrease in the occlusal vertical dimension (OVD) were noted (Figure 1C, D).

A panoramic radiograph revealed a thin layer of enamel in most erupted teeth (Figure 1E). The right and left upper canines, the left lower canine and the lower right, and left second and third molars were unerupted. However, the upper right and left second molars and the lower left first premolar were agenesic. The lower right first and second premolars were endodontically treated. Cephalometric analysis revealed a skeletal Class III relationship with maxillary retrusion and normal mandibular position (Figure 1F).

Taking into account anamnesis as well as the clinical and radiographic findings, diagnosis of hypoplastic AI was made. The family history revealed that the patient's younger brother also had similar dental abnormalities. The inheritance pattern was autosomal recessive.

**Figure 2:**
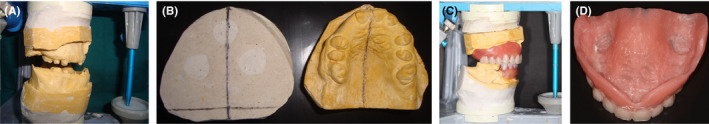
Preoperative preparations. A, Lateral view of diagnostic articulation in the correct vertical dimension showing a 3 mm space between the maxillary and mandibular arches and confirming the interarch Class III relation. B, Preoperative simulation on maxillary casts showing the allowed amount of maxillary protrusion (7 mm). C, Artificial teeth arranged in advanced position. D, Maxillary and mandibular overdentures

Casts made from irreversible hydrocolloid impressions were positioned in the centric relationship using occlusal rims adjusted to the patient's correct OVD. The mounted diagnostic casts showed an interarch sagittal gap and a 3 mm vertical space between maxillary and mandibular teeth at the correct OVD (Figure 2A).

**Figure 3:**
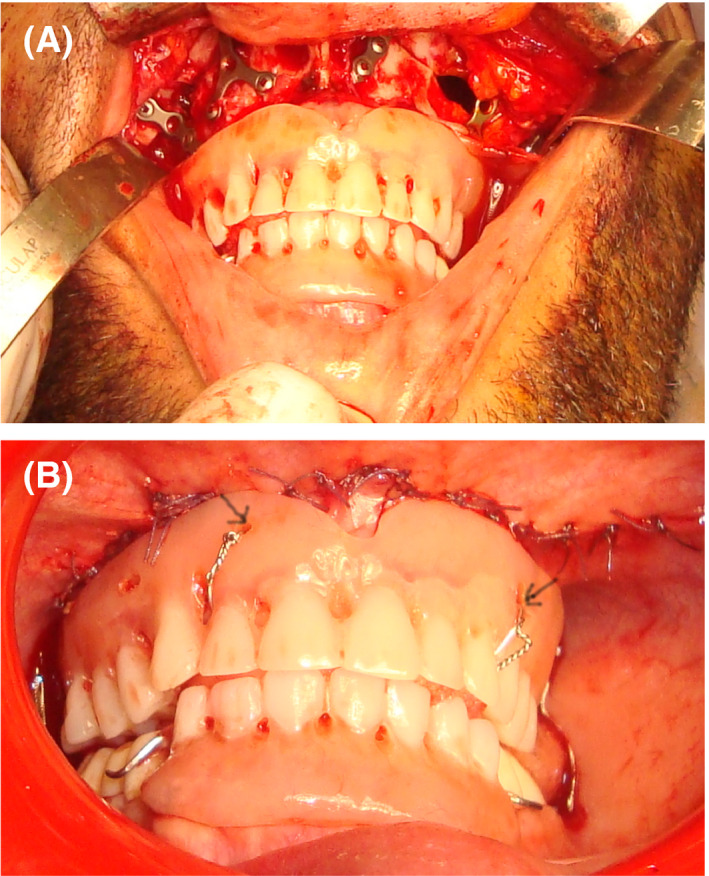
Lefort type I osteotomy surgery. A, Maxillary bone fixation in the advanced position using titanium plate fixation during Lefort type I osteotomy surgery. B, The upper complete overdenture was fixed to the maxilla using metallic wires (Black arrow)

**Figure 4:**
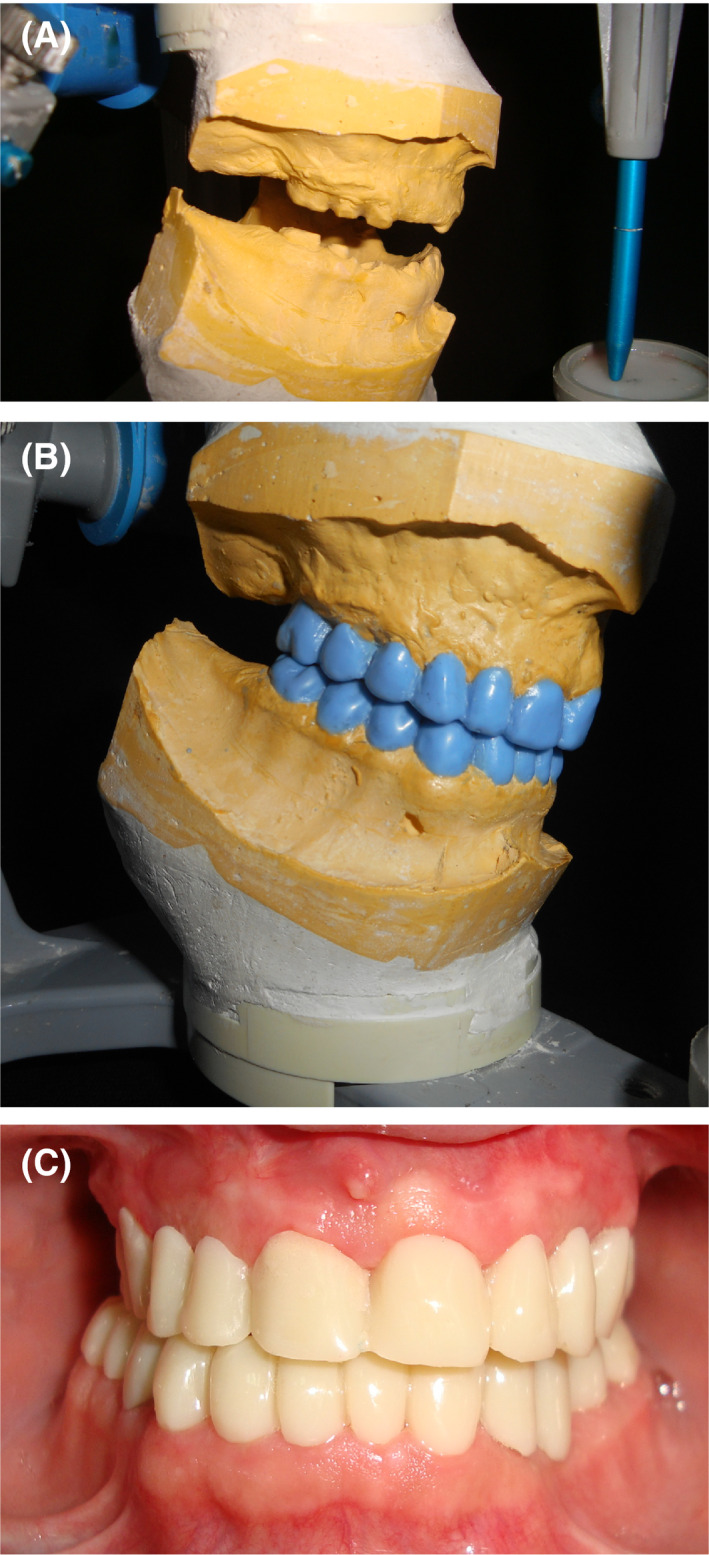
Provisional crowns. A, Postsurgery articulated casts showing the improvement of the interarch Class III relation. B, Diagnostic wax‐up. C, Laboratory‐processed provisional crowns prepared with the aid of the diagnostic wax‐up

**Figure 5:**
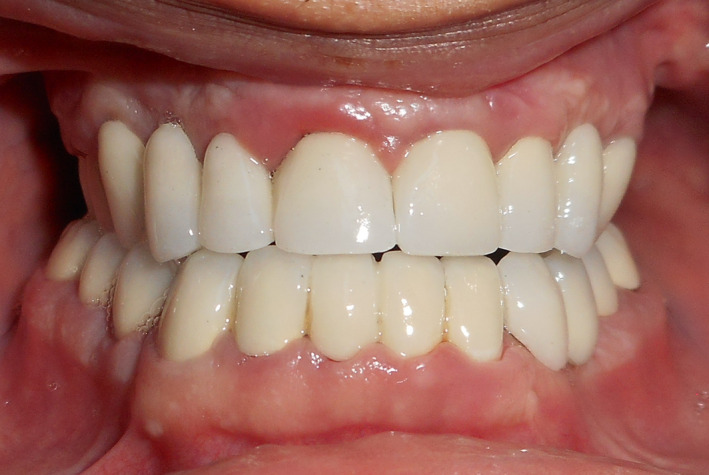
Postoperative view of the cemented prostheses. A, Front view of the cemented metal‐ceramic prosthesis

To correct the skeletal Class III, orthodontic treatment associated with orthognathic surgery were proposed. However, orthodontic treatment was not possible because of the lack of anchorage. The correction of the skeletal Class III via only an orthognathic surgery approach (Lefort I osteotomy with maxillary advancement) was then chosen. The decision was to perform a 7 mm maxillary advancement. Thus, a preoperative simulation of the maxillary protrusion was performed on the maxillary cast (Figure 2B). Complete maxillary overdenture and mandibular removable partial denture were prepared on the modified mounted diagnostic casts (Figure 2C). The removable dentures (Figure 2D) were used to guide maxillary advancement during surgery. In fact, the Lefort I osteotomy was carried out and the maxilla was moved forward until a perfect contact between the dentures was found. Then, the maxilla was fixed in this position using titanium plates (Figure 3A). The upper complete overdenture was fixed to the maxillary bone using metallic wires to ensure its healing in the new anterior position (Black arrow Figure 3B). The removable dentures were kept in mouth for 3 months. Later, postsurgery casts were mounted and the improvement of the interarch relationship was assessed (Figure 4A). Wax‐ups were performed (Figure 4B). Next, preparation for complete crowns was performed. Provisional crowns were made with acrylic resin according to the wax‐up (Figure 4C). The gingival and crown lengthening surgery for the maxillary and mandibular anterior teeth was performed to improve the crown height and therefore enhance esthetics. Teeth preparations were adjusted in the cervical parts, and the provisional crowns were rebased to fit the new cervical limits. Then, provisional crowns were manufactured in the laboratory and tested on the patient for 3 months to validate the new OVD. The patient showed good adaptability to the new occlusion and was satisfied with the esthetic and functional outcome. The temporomandibular joints and mastication muscles showed no abnormality following the new occlusion. An irreversible hydrocolloid impression of the provisional crowns was obtained to enable casts to serve as a guide for the production of definitive metal‐ceramic crowns (Figure 5A). The patient was comfortable and satisfied with the treatment results. A regular follow‐up every 6 months was performed during 5 years. The treatment results were stable and satisfying.

## 3 DISCUSSION

Amelogenesis imperfecta (AI) can be diagnosed based on clinical and radiographic examinations.^8^ AI type 1 (Hypoplastic type) is characterized by an enamel thickness reduction. In addition, radiographs usually display thin enamel laminate on slim appearing teeth, where the proximal contacts are often missing.^9^, ^10^ AI could be associated with skeletal abnormalities, such as the Class III malocclusion.^11^

The treatment strategy differs depending on the patient's age. In this case, the patient is an adult (22 years old). So, the treatment approach is definitive because it is not limited by the cranio‐facial development. The treatment strategy of young patients with A.I must be progressive in order to give way to cranio‐facial growth.^12^

Our treatment objectives for this patient were to correct the skeletal malposition and to adapt a reorganized approach, involving different disciplines.^11^

To correct Class III malposition, presurgical orthodontics is often unachievable with AI patients because of the absence of teeth or short crown height that leads to anchorage failure.^13^ Furthermore, poor enamel condition affects and limits the resin composite survival on the A.I crowns.^14^ Therefore, the surgical approach used in this case was Lefort I osteotomy advancement. This procedure requires a collaboration with the oral and maxillofacial surgeon to decide on the suitable amount of maxillary protrusion allowed during the surgery, which was 7 mm in this case, and to guide the maxilla in the correct advanced position during the surgery. Thus, overdentures that were prepared on modified casts to simulate maxillary advancement and that were fixed to the maxilla bone were a smart way to allow the correct maxilla positioning and to insure its strengthening in the advanced position.

AI is often associated with OVD loss because of the fast tooth wear.^15^ OVD restoration and esthetic improvement are the most important goal for prosthetic rehabilitation. Therefore, in the present case, an increase of the patient's OVD to the correct position using provisional crowns was performed. The provisional crowns were kept in mouth for 3 months to assess the patient's adaptability to the newly established OVD and to the new occlusal scheme.^16^ It is reasonable to assume that there is an optimal adaptative space concerning OVD, and not a fixed point. So, it is possible to change it in both directions. In fact, therapeutic OVD should be in harmony with all the anatomical and neuro‐physiological determinants.^17^ Indeed, the preservation of an interocclusal space in resting posture and the lack of contact between the dental arches during oral functions as phonation are among the targets of therapeutic OVD that are as important as esthetics and lip competency.^17^ A long follow‐up is needed to check and reevaluate these functions and to ensure patients’ satisfaction.

## CONFLICT OF INTEREST

None.

## AUTHOR CONTRIBUTIONS

A.L, S.B, and Y.M: contributed to the prosthetic treatment of the case, and prepared and drafted the manuscript; J.BM: contributed to the periodontal treatment of the case; M.O: contributed to the oral and maxillofacial surgery of the case; S.GM and L.M: reviewed the manuscript.

## Data Availability

The authors confirm that all relevant data of this case report are available from within the paper.
